# Emergency presentation of colorectal cancer in Africa: a scoping review

**DOI:** 10.1136/bmjopen-2025-110931

**Published:** 2026-01-22

**Authors:** Simbarashe Chinyowa, Grant Murewanhema, Leolin Katsidzira, Jennifer Moodley, Mike Chirenje, Godfrey Muguti

**Affiliations:** 1 Surgical Sciences, University of Zimbabwe Faculty of Medicine and Health Sciences, Harare, Harare, Zimbabwe; 2 University of Zimbabwe Faculty of Medicine and Health Sciences, Harare, Harare Province, Zimbabwe; 3 School of Public Health, University of Cape Town Faculty of Health Sciences, Cape Town, WC, South Africa; 4 Department of Obstetrics, Gynaecology & Reproductive Sciences, University of California San Francisco School of Medicine, San Francisco, California, USA; 5 Faculty of Medicine & Health Sciences, University of Zimbabwe, Harare, Zimbabwe

**Keywords:** Colorectal surgery, Africa South of the Sahara, Risk Factors, Disease Management, Gastrointestinal tumours, Epidemiology

## Abstract

**ABSTRACT:**

**Objectives:**

This review identified and mapped the current evidence on emergency presentation of colorectal cancer in Africa.

**Design:**

Scoping review based on methodology by Arksey and O’Malley.

**Data sources:**

Medline (via PubMed), Embase and African Journals Online were searched between 29 January 2025 and 6 March 2025.

**Eligibility criteria:**

Studies published in English and describing the burden, risk factors and management options for emergency presentation of colorectal cancer in Africa were included. Full-text articles of all epidemiological study designs were considered with no publication date limit. Grey literature such as conference abstracts and letters to the editor were considered if sufficient study detail was provided to map data to the research questions.

**Data extraction and synthesis:**

Two independent reviewers extracted data from the included studies onto a standardised electronic form. Data were narratively synthesised using predefined themes.

**Results:**

Twenty-seven studies are included in this review. There was wide variability in reported emergency presentation colorectal cancer rates (8.3% to 64.9%). There were few data on early mortality, and none on long-term outcomes following surgery for colorectal cancer. Emergency presentation of colorectal cancer was characterised clinically by bowel obstruction, bowel perforation and peritonitis. Only one study reported on risk factors for emergency presentation of colorectal cancer. Use of diagnostic modalities was variable and depended on resource availability. Surgery was the mainstay of treatment. Endoscopic stenting was only available as a treatment option in South Africa.

**Conclusions:**

Emergency presentation of colorectal cancer is a significant clinical, oncological and health system metric, yet there is a dearth of evidence on the subject in the African context. Available evidence demonstrates the need for more studies on emergency presentation of colorectal cancer, focused on risk factors, prevalence, treatment options and short-term and long-term outcomes.

**STRENGTHS AND LIMITATIONS OF THIS STUDY**

- This scoping review was conducted according to an evidence synthesis methodology and was reported using the Preferred Reporting Items for Systematic Reviews and Meta-Analyses extension for Scoping Reviews tool.
- This review was limited to publications in the English language only and may bias our findings since some African countries are French and Portuguese speaking.
- While a comprehensive literature search was made, it is possible that some relevant articles may have been missed, especially those not available online.
- As this was a scoping review, we did not make a formal appraisal of the strength of evidence of each study.

## INTRODUCTION

 Colorectal cancer (CRC) incidence and mortality are declining in some high Human Development Index (HDI) countries, while, almost unnoticed, it has been increasing in lower HDI countries.^1^ Specifically, in Africa, CRC was previously considered to be rare, but the estimated increase in age-standardised incidence rate between 2010 and 2019 is 11%.^2^ This has been attributed to an increasingly ageing population, environmental, dietary and lifestyle factors.^2^,^4^ Notably, incidence and mortality rates in Africa are likely under-reported, as there are only 35 population-based cancer registries across 25 African countries out of 54 countries on the continent.^5^

In addition, no country in Africa offers organised population-based CRC screening.^6^ Consequently, a significant proportion of CRC is diagnosed at late stages (stage 3 and 4). For example, in a study of 11 population-based registries in sub-Saharan Africa, 31% of CRC cases, between 2011 and 2015, were diagnosed at stage 4.^7^ In sub-Saharan Africa, the overall 5-year survival is approximately 48% and highly variable between different countries.^8^ By comparison, the estimated 5-year survival in the USA is about 64%.^1^ In addition to oncological implications, late-stage CRC may present clinically with complications requiring emergency intervention. For some patients, this emergency presentation is the first opportunity for diagnosis of the cancer. An emergency presentation of cancer is defined as the diagnosis of cancer during an unscheduled or unplanned hospital admission.^9^ Emergency presentation CRC (EP-CRC) rate is the number of emergency presentations as a proportion of the total number of CRC cancer diagnoses over a defined period. EP-CRC rate is reported between 10% and 30% in HDI settings.^10^,^12^ Multiple factors may contribute to EP-CRC, including host factors, tumour characteristics and other factors such as ethnicity, socioeconomic status, distance from hospital and seasonal variability.^12^ Some of these factors may be modifiable and present an opportunity for interventions aimed at reducing the EP-CRC rate.

EP-CRC may be used as a performance indicator, a potential measure of diagnostic delays, prolonged referral pathways and missed opportunities.^13^ EP-CRC frequently requires operative intervention and forebodes a significantly poorer perioperative mortality, overall survival and cancer-specific survival.^14^ In a landmark Scottish study, Anderson *et al.* reported perioperative mortality was 9% in patients who had elective surgery for CRC versus 19% in patients who had emergency surgery.^15^ In another study, McArdle *et al* found that even long-term outcomes were adversely affected, with 5-year survival of 39.1% in emergency surgery for CRC versus 57.5% for elective CRC patients.^16^ This pattern has been subsequently confirmed by several others,^17^,^19^ underscoring the challenges of CRC treatment in an emergency setting.

Given the limited cancer screening, symptom awareness and treatment access in Africa, there likely is a greater proportion of EP-CRC. However, the majority of studies on EP-CRC to date have been done in high HDI settings. Data from African countries concerning EP-CRC are sparse. The burden of EP-CRC in Africa is unclear, the contributory factors unknown, and the management strategies employed unascertained. This scoping review aimed to identify and map literature on EP-CRC in Africa. It is anticipated that the results of this scoping review will highlight areas for research and inform policymakers on the burden and significance of EP-CRC. This may prompt innovative approaches to address the needs of this key subset of CRC patients as well as to reduce the incidence of EP-CRC.

## Methods

### Search strategy and selection criteria

This scoping review aimed to summarise the literature and identify gaps concerning EP-CRC in Africa. Specifically, we sought to answer the following questions:

- What is the burden of EP-CRC in Africa?
- What are the risk factors associated with EP-CRC in Africa?
- What are the clinical management options employed for EP-CRC in Africa?

We followed five steps originally outlined by Arksey and O’Malley.^20^ The Population, Concept and Context framework^21^ was used to map studies to the research questions. Results were reported in line with the Preferred Reporting Items for Systematic Reviews and Meta-Analyses extension for Scoping Reviews (PRISMA-ScR).^22^ Eligible patients were those in whom a first diagnosis of colon or rectal cancer was made in an emergency context. Studies not focused solely on EP-CRC were also considered, provided information relating to EP-CRC was reported. Studies involving interventions in previously diagnosed CRC patients were excluded. This review only considered studies conducted in Africa, published in English and full-text articles of all epidemiological study designs including case reports, cohort studies, case–control studies and systematic reviews. Grey literature such as conference abstracts and letters to the editor were considered if sufficient study detail was provided to map data to the research questions.

A preliminary search of PubMed was conducted to identify studies on EP-CRC. Key terms appearing in the titles and abstracts of identified articles were used to build a search strategy for PubMed. Medical Subject Headings identifying these articles were also incorporated into the search (online supplemental appendix A). A medical librarian specialist (ASN) was consulted as part of the process of developing the search strategy. The PubMed search strategy was adapted to conform to the syntax used for the other included databases. Between 29 January 2025 and 6 March 2025, a comprehensive literature search was made on PubMed, African Journals Online and Embase to identify literature meeting the inclusion criteria. There was no publication date limit for inclusion of the studies. Once articles to be included in the review were identified, their reference lists were screened for additional relevant papers and where identified, these articles were located by a manual search.

All studies identified through the database searches were uploaded into Rayyan software.^23^ Duplicate studies were identified and removed. A two-step process was followed in screening for relevant studies. In the first step, SC and GM independently performed a title and abstract screen to identify articles meeting the inclusion criteria. Each study was flagged as ‘include’, ‘exclude’ or ‘maybe’. The two independent lists were then compared. In the event of disagreement, this was resolved by internal consensus. The second step consisted of a full-text search for articles tagged as ‘include’ and ‘maybe’ in step 1. The retrieved articles were again independently reviewed by SC and GM against the study inclusion and exclusion criteria. Any disparities in article selection were resolved by consensus. Where an article was tagged as ‘exclude’ at this stage, the reason was recorded and documented in the final report (online supplemental appendix B).

SC and GM extracted data from each of the included studies onto a standardised electronic form (online supplemental appendix C). The two reviewers resolved any disagreements in the abstracted data by discussion.

### Data analysis

Extracted data included study identification data, sample size, methodological characteristics, emergency presentation rate, intestinal obstruction rate, risk factors and clinical interventions. A narrative summary of included studies was made, including an overview of the number and characteristics of studies meeting inclusion criteria. Where possible, quantitative evidence was aggregated using summary statistics. Significant gaps in the literature were highlighted and discussed.

As this is a scoping review of a sparsely documented phenomenon, eligible studies were considered regardless of the strength of evidence. The intention was to map out the available evidence rather than to rigorously critique it as required for a systematic review.

### Role of the funding source

The funder had no role in the study design, data collection, data analysis, data interpretation or writing of the report.

### Patient and public involvement

Patients or the public were not involved in the design, or conduct, or reporting, or dissemination plans of our research.

## Results

**Figure 1:**
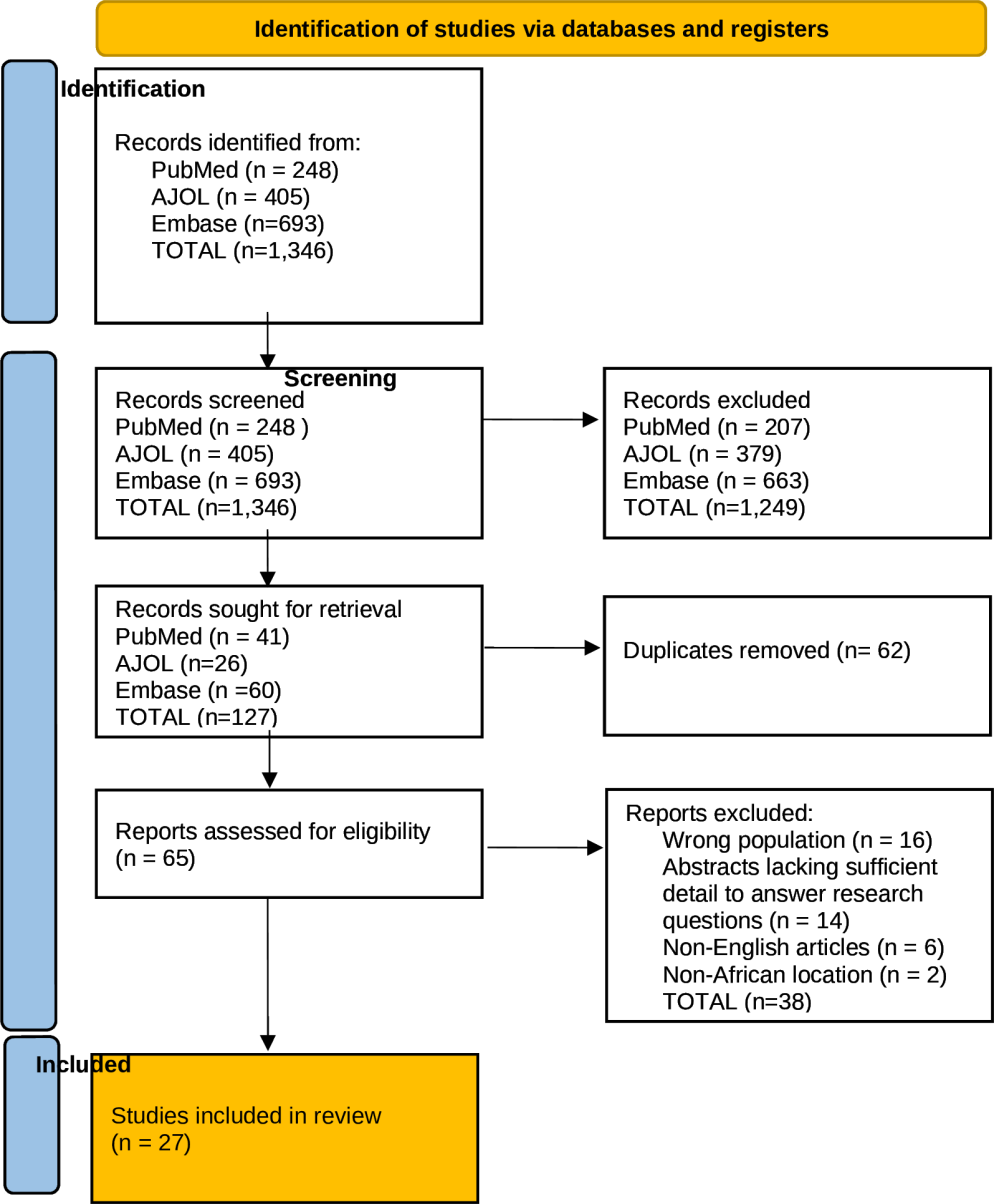
PRISMA flowchart of the study selection process. Source: Page MJ, et al. BMJ 2021;372:n71. doi: 10.1136/bmj.n71. PRISMA, Preferred Reporting Items for Systematic Reviews and Meta-Analyses.

The literature database search yielded a total of 1346 records. These were screened by title and abstract and resulted in the exclusion of 1249 records. Of the remaining 127 records, 62 duplicates were removed. The resulting 65 records were reviewed against the predefined eligibility criteria. After excluding a further 38 records for various reasons, 27 studies were included in the final review. The PRISMA flowchart detailing the study selection process and reasons for exclusion is shown in figure 1.

### Preferred Reporting Items for Systematic Reviews and Meta-Analyses

Online supplemental table 1 describes the characteristics of the 27 studies published between 1996 and 2024. The majority (14) were retrospective studies, with the remainder distributed as follows: prospective studies (6), cross-sectional studies (2), case reports (4) and letters to the editor (1). Three studies reported on colon cancer only,^24^,^26^ another three on rectal cancer only^27^,^29^ and the majority, 21, reported on both colon and rectal cancers.^30^,^49^ Two studies included patients with cancer of the anus together with colon and/or rectal cancer patients.^35 50^ Patient ages ranged from 10 to 99 years. The following countries (number of studies) were represented: Kenya (3), Nigeria (9), Tanzania (2), Ethiopia (2), Mozambique (1), South Africa (6), Egypt (2), Ghana (1), Sudan (1), Rwanda (1).

Only one study focused primarily on EP-CRC,^34^ and five other studies reported on EP-CRC rate as part of other outcomes.^28 35 39 45 51^ In the remaining 21 studies, EP-CRC could be inferred from data on patients diagnosed with CRC presenting with intestinal obstruction or intestinal perforation. While several studies reported on colorectal bleeding, it was not possible to distinguish between cases managed emergently or electively; therefore, these data were not included in the assessment of EP-CRC.

There is wide variability in the reported EP-CRC rate, being lowest (8.3%) in a study by Rahman *et al*^28^ in Nigeria and highest (64.9%) in a study by Herman *et al*^46^ in Tanzania. The average EP-CRC rate from these published studies was calculated as 32.3% (409/1267 cases) (see online supplemental table 2).

The intestinal obstruction rate is lowest (9%) in a study by Zeeneldin *et al*^37^ in Egypt and highest (53.3%) in a study by Abudu *et al*^47^ in Nigeria. The calculated average intestinal obstruction rate is 18.9% (1133/5988 cases) (see online supplemental table 3).

Few studies reported on early mortality following EP-CRC surgery compared with elective CRC surgery. In these studies, mortality was consistently higher among the emergency group as follows: Saidi *et al,* emergency 28% versus elective 17%^32^; Aderibigbe *et al,* emergency 23.2% versus elective 9.1%^34^ and Rahman *et al,* emergency 5.9% versus elective 0.0%.^28^

Only two of the 27 studies reported on the risk factors associated with EP-CRC. In a study in Nigeria, Aderibigbe *et al* described risk factors for EP-CRC.^34^ On univariate analysis, factors contributing to EP-CRC included patient-reported delays such as reassurance from healthcare workers, use of self-medication, belief that symptoms were not concerning and financial barriers. Disease factors included metastatic disease, higher clinical stage and tumours proximal to the splenic flexure. Other risk factors identified were lower education levels, lower household income, non-use of motorised transport to get to hospital and hospital site. However, on multivariate analysis, factors that remained associated with higher odds of EP-CRC were metastatic disease (stage IV), a lower household income and hospital site, being more frequent at a particular referral-level hospital compared with others. In a case report, Mahabane *et al* suggested that pregnancy may contribute to EP-CRC by masking symptoms of CRC.^26^

Online supplemental table 4 summarises the clinical management methods employed in EP-CRC. It illustrates the wide range of workup and treatment options documented in the various studies included. Following history, physical examination and intravenous fluid resuscitation, there was variable use of radiological imaging and endoscopy in diagnosis and treatment. Imaging investigations employed included plain abdominal X-rays, ultrasonography, as well as cross-sectional imaging such as CT scan and MRI.^24^,^2729 30 32 34 36 41 47 51^ In most cases, imaging use was done in a stepwise fashion, with plain radiography or ultrasound used first, followed by CT or MRI, where available, to further characterise the cancer and assess for metastases. The use of CT was not universally available in all settings; for example, Saidi *et al* report the increased availability of CT and colonoscopy as an adjunct for tumour evaluation over a 13-year period.^32^ Other diagnostic modalities used included colonoscopy, sigmoidoscopy, transrectal ultrasound and double-contrast barium enema.^47 52^ In a few studies, exploratory laparotomy was used with both diagnostic and therapeutic intent.^31 34^

Studies that reported treatment of CRC without discriminating between elective CRC cases and EP-CRC were not included in this analysis. Thirteen studies reported clinical interventions related specifically to EP-CRC.^2426 27 29 31 33 34 36 41 50 53^,^55^ Interventions included surgical procedures,^2426 27 29 31 33 34 41 50 53^,^55^ endoscopic stenting^27 36 41^ and non-surgical treatment in patients not eligible for surgery.^36 53^ Surgical procedures largely depended on tumour location, staging and patient clinical condition. The range of procedures included segmental resections, Hartmann’s procedure, low anterior resection and abdominoperineal resection,^2426 27 29 31 41 53^,^55^ bowel diversion (ostomies)^2426 27 29 33 41 50 53^,^55^ and bypass procedures.^27 54^ It was apparent that the goals of surgery were either curative or palliative, though this distinction was not explicitly stated in most studies. Two studies did not detail the type of surgery performed, though they indicated it was emergency surgery.^33 34^

Only three studies reported on stenting as an intervention for EP-CRC, all from South Africa.^27 36 41^ Stenting was employed as a bridge to surgery and as palliation. However, not all stenting efforts were successful, and failed attempts were rescued by surgery or appropriate palliation if unfit for surgery.^27 36^ Patients older than 60 years and patients with right-sided tumours were more likely to be managed by stent placement.^27^

Aderibigbe *et al* observe that patients who presented emergently were more likely to receive surgery and less likely to receive chemotherapy, compared with patients who presented electively.^34^ Other studies^25 26 33 41 50^ reported the use of adjuvant chemotherapy and/or radiotherapy, though only in passing detail. The case report by Mahabane *et al* illustrates that treatment was individualised, with medical termination of pregnancy being necessary alongside oncological treatment.^26^

## Discussion

Appreciating EP-CRC gives insight into the quality and efficiency of CRC care services. This review summarises the available evidence on EP-CRC in Africa. It highlights the dearth of studies focused on EP-CRC, a wide variability in EP-CRC rates, and identifies gaps in the available literature pertaining to risk factors, management methods employed and outcome data relating to EP-CRC.

Six studies defined cases as EP-CRC. However, there was variability in the conceptualisation of what constitutes an emergency. The different concepts on which these definitions were based included timing of admission, route of admission and clinical picture. For example, Aderidigbe *et al* defined emergency cases as those diagnosed in an unplanned admission (timing).^34^ In contrast, Selemane *et al* considered patients admitted via the emergency rooms as emergencies (route of admission).^35^ To add another layer, Dakubo *et al* and Uwamariya *et al* based the categorisation as emergency on the presenting features (clinical picture) while Rahman *et al* did not explicitly define which cases were considered emergencies.^28 39 45^ This review identified a further 21 studies in which the term ‘emergency presentation’ was not used, but intestinal obstruction and intestinal perforation patients were surgically treated as EP-CRC (clinical picture).

The different conceptual approaches result in small but significant differences in inclusion and exclusion criteria. A few examples are described for clarity. (1) A patient with partial intestinal obstruction or contained perforation may allow for clinical stabilisation, full workup and ultimately, surgery as an ‘elective case’ but the same patient may have been admitted via the emergency room, and as an unplanned admission and categorised as ‘emergency’. (2) A patient may present with significant anaemia, requiring unplanned admission and transfusion. Underlying CRC is then diagnosed, fully worked up and treated by chemoradiotherapy. This patient could be classified as an emergency presentation based on timing and admission route, but if the inclusion criteria focused only on the clinical picture, then the patient may be difficult to categorise. Furthermore, if the focus is on ‘emergency surgery’, this patient would not be included. Literature alludes to the difficulty in having a single definition of emergency cancer presentation and this will likely continue to be an evolving concept.^10 51 52 56^

The EP-CRC rate is a health system performance metric, particularly reflecting efforts in cancer screening, diagnosis and treatment.^53^ This review showed a wide range in EP-CRC rate, ranging from 8.3% to 64.9%. Even allowing for the different underlying study populations and varying time points when the studies were conducted, these rates are high compared with those in HDI settings, which range between 10% and 30%.^10 12^ The higher rates in Africa likely reflect the lack of population screening and various barriers to timely diagnostic and treatment services.^6^ Diagnostic delay is a complex process involving the interplay of patient factors, healthcare provider and system factors, and disease factors.^54^ These factors require exploration through appropriately designed studies. The current evidence shows a dearth of African studies reporting on risk factors associated with EP-CRC and ways to prevent these emergencies. This is a significant gap requiring more research as Africa, which is considerably large geographically, has extensive diversity in population and level of development across nations and within individual countries. In a single study, metastatic disease, lower household income and hospital site were shown on multivariate analysis to be significant risk for emergency presentation.^34^ Together, these risks fall under disease, patient and system factors according to the Model of Pathways to Treatment refinement of the Andersen Model of Total Patient Delay.^54^ Disease factors likely cannot be modified, but efforts to implement screening, improve health-seeking behaviour and minimise system diagnostic inefficiencies may decrease the rate of emergency presentations. This is critical because EP-CRC has been shown to result in worse short-term and long-term outcomes.^9^

Optimal evaluation of CRC requires colonoscopy to evaluate the entire colon, biopsy of the tumour for histology and imaging for local and distant involvement (CT, MRI, Endoluminal ultrasound for rectal tumours and PET-CT in selected cases). The current evidence shows a variability in the use of radiological imaging and endoscopy in diagnosis, dependent on availability. Few centres had access to colonoscopy and CT/MRI services. There was no radiological support in some centres, with exploratory laparotomy necessary following clinical evaluation. This has obvious disadvantages in a cancer setting. Limited diagnostic evaluation means that information such as the presence of tumour, site of tumour and extent of tumour is not available preoperatively. Therefore, the ability to plan ahead is severely curtailed, with morbidity and mortality implications.

Surgery was the mainstay of treatment in all studies in this review. Surgical procedures depended on tumour location, tumour staging and patient clinical condition. This is in keeping with standard practice elsewhere in the world.^55^ However, only a few studies reported short-term outcomes,^28 32 34 41^ and none reported long-term outcomes following surgery. In the short term, mortality following surgery reflects patient, disease and health system factors. These are even better analysed when contrasted against elective surgery outcomes. In Africa, early mortality following emergency surgery is higher than that following elective surgery similar to findings in other parts of the world.^15 57^ However, mortality is higher than that reported in HDI countries.^57^ Unfortunately, loss to follow-up is one of the biggest challenges to assessing long-term outcomes following treatment.^39^ This challenge is particularly amplified in low-resource settings such as Africa, with barriers to consistent health record keeping.

Three South African studies reported on stenting as an intervention for EP-CRC.^27 36 41^ Endoscopic stenting allows for stabilisation of the patient and planning for safer, elective surgery at a later date (bridge-to-surgery).^58^ Stenting is also indicated as palliation in those patient who are unsuitable for surgery.^59^ The very limited use of stenting in the current review likely reflects the limited capacity for endoscopic services in Africa.^60^ However, it is worthwhile noting that stenting is not without its challenges. While stenting is well established for left-sided tumours, further research is required to validate its use in the right colon.^58^ Additionally, complications such as stent migration, reobstruction and perforation may limit its use. Another caution to the use of stents arises from concerns that disease recurrence may be higher in patients who are stented as a bridge to surgery, compared with emergency surgery.^59 61^

In this review, few studies reported the use of adjuvant chemotherapy and/or radiotherapy following surgery for EP-CRC. Patients who presented emergently were less likely to receive adjuvant therapy compared with elective patients. Cancer therapy, in an elective setting, allows for a thorough work-up, including staging, and the convening of a multidisciplinary team to discuss the best treatment for each patient. EP-CRC takes away the privilege of time to plan and imposes surgery or stenting as the most effective modalities for relief of the immediate cancer complication. Other factors contributing to limited use of adjuvant therapy following surgery include surgical convalescence, lack of access to chemotherapy and radiotherapy, limited survival, loss to follow-up, personal and societal attitudes to adjuvant therapy.^62 63^ This is an area requiring further study in Africa to elucidate these factors.

While our review adds significantly to the body of knowledge of EP-CRC, there are several limitations to it. First, we only considered articles whose full text is available in the English language, yet a proportion of African countries are French-speaking, with a few Portuguese-speaking African countries. Therefore, it is possible that some articles may have been omitted. Second, the studies included were heterogeneous in terms of design and sample size, for example, in some studies, CRC data were contained within studies focused on non-cancer pathologies such as other causes of large bowel obstruction. A major limitation of our study, as with most scoping reviews, is that we did not make a formal appraisal of the strength of evidence of each study. However, this was inevitable, given the sparse distribution of data on the continent.

In conclusion, EP-CRC is a significant clinical, oncological and health system metric, yet there is a dearth of evidence on the subject in the African context. However, there is sufficient evidence to advocate for increasing access to diagnostic endoscopic and cross-sectional imaging services, and for strategies to increase awareness among the population and healthcare workers. These interventions must be implemented in tandem with more studies on this essential health metric, with an emphasis on contributory factors, short and long-term outcomes.

## Supplementary material

10.1136/bmjopen-2025-110931 online supplemental file 1

## Data Availability

Data sharing is not applicable as no datasets generated and/or analysed for this study. All data relevant to the study are included in the article or uploaded as supplementary information.
