# Supplementary material for: Emergency presentation of colorectal cancer in Africa: a scoping review
Source: BMJ Open. 2026 Jan 22;16(1):e110931. doi: 10.1136/bmjopen-2025-110931 (PMC12829401; doi:10.1136/bmjopen-2025-110931)
Supplement: online supplemental file 1 [file bmjopen-16-1-s001.docx]

**Supplemental Table 1: Characteristics of included studies**

| **Publication details** | **Methodology** | | | | **Main findings** | |
| --- | --- | --- | --- | --- | --- | --- |
| **Author and Year of publication**  **Country** | **Study design** | **Cancer site** | **Study focus and sample size** | **Definition of Emergency Presentation** | **Epidemiology of**  **EP-CRC* and IO rate**† | **Risk factors for EP-CRC** |
| **Abdulkarim et al.**  **2021**  **Kenya** | Letter to editor | Colon  Rectum | Clinicopathological characteristics of patients with CRC  **249** | Clinical presentation | IO rate 26·2%(65/249) | NR |
| **Ugwu et al.**  **2001**  **Nigeria** | Retrospective | Colon  Rectum | Adult intussusception  **22** | Clinical presentation | NR | NR |
| **Herman et al.**  **2017**  **Tanzania** | Case report | Colon | Schistosomiasis and colon cancer  **1** | Clinical presentation | Obstruction due to sigmoid lesion with schistosoma mansoni and colon adenocarcinoma | NR |
| **Saidi et al.**  **2008**  **Kenya** | Retrospective | Colon  Rectum | Presentation, treatment and outcome pattern of patients with CRC  **259** | Clinical presentation | IO rate 16·9%  Mortality rate: Emergency surgery 28% (10/36)  Mortality rate: Elective surgery 17% (29/167) | NR |
| **Zingeta et al.**  **2023**  **Ethiopia** | Prospective | Colon  Rectum | Outcomes and prognostic factors of patients with CRC  **209** | Clinical presentation | IO rate 26·8% (56/209) |  |
| **Aderibigbe et al.**  **2024**  **Nigeria** | Prospective | Colon  Rectum | Risk factors for EP-CRC  **535** | Unplanned admission  And  Clinical presentation | EP-CRC rate 30·7% (164/535)  IO rate 16·7% (90/535)  30-day mortality rate:  Emergency presentation 23·2%  Elective presentation 9·1% | Metastasis at the time of diagnosis;  Reassurance from a health-care provider;  Use of self-medication;  Belief that symptoms were not concerning;  Financial barriers;  Tumours proximal to the splenic flexure;  Lower education levels;  Lower median household monthly income;  Walking to hospital rather than using motorized transport;  Hospital site; |
| **Selemane et al.**  **2001**  **Mozambique** | Retrospective | Colon  Rectum  Anus | Disease profile of patients with CRC and anal cancers  **62** | Admission via emergency room | EP-CRC rate 50·8% (32/62) | NR |
| **Moolla, Z et al.**  **2014**  **South Africa** | Retrospective | Colon  Rectum | Demographics and management of patients with obstructing CRC  **203** | Clinical presentation | IO rate 14·3% (203/1425) | NR |
| **Warden et al.**  **2013**  **South Africa** | Prospective | Colon Rectum | Safety of Self-Expanding Metal Stents in left-sided obstructing CRC without peritonitis or perforation  **77** | Clinical presentation | All patients were obstructed.  Successful stent 78% (60/77)  Bridge to surgery 25/60  Palliation 35/60 | NR |
| **Zeeneldin et al.**  **2011**  **Egypt** | Retrospective | Colon  Rectum | Effect of age on treatments and outcomes of CRC patients in a population based registry  **159** | Clinical presentation | IO rate 9% (14/159) | NR |
| **Iliyasu et al.**  **1996**  **Nigeria** | Retrospective | Colon  Rectum | Changes in pattern of CRC over 20 years  **526** | Clinical presentation | IO rate 13·9% (73/526) | NR |
| **Dakubo et al.**  **2010**  **Ghana** | Prospective | Colon  Rectum | New cases of CRC  **359** | Clinical presentation | EP-CRC rate 19·8% (71/359) | NR |
| **Rahman**  **2010**  **Nigeria** | Prospective | Rectum | Incidence, presentation, diagnosis, and treatment outcomes of rectal cancer  **36** | Not clearly defined | EP-CRC rate 8·3% (3/36)  30-day Operative mortality rate  Emergency (2/34) 5·9%  Elective (0/34) 0·0% | NR |
| **Montwedi**  **2019**  **South Africa** | Case report | Rectum | Rectal prolapse  **1** | Clinical presentation | Intestinal Obstruction due to a prolapsed rectal tumour | NR |
| **Soressa et al.**  **2016**  **Ethiopia** | Cross-sectional | Colon  Rectum | Prevalence, causes and management outcomes of intestinal obstruction  **262** | Clinical presentation | Colonic tumours were 5·3% of all intestinal obstructions | NR |
| **Ayandipo et al.**  **2013**  **Nigeria** | Retrospective | Rectum  Anus | Demographic, clinical features, management and outcomes of patients undergoing abdominoperineal resection for rectal and anal tumours  **61** | Clinical presentation | IO rate 20% (12/61) | NR |
| **Ndlovu N et al.**  **2023**  **South Africa** | Retrospective | Colon  Rectum | Clinicopathological spectrum and management of obstructed CRC  **510** | Clinical presentation | IO rate 20·4% (510/2504)  Mortality rate 47·5% (242/510) | NR |
| **Udo IA et al.**  **2023**  **Nigeria** | Cross-sectional | Colon  Rectum | Causes of intestinal obstruction  **70** | Clinical presentation | NR | NR |
| **Norman J et al.**  **2023**  **South Africa** | Retrospective | Colon  Rectum | Large bowel perforation amongst CRC patients  **94** | Clinical presentation | Large bowel perforation 3·7% (94/2523)  IO rate 35·1% (33/94) | NR |
| **Ali et al.**  **2023**  **Sudan** | Case report | Colon | Intussusception of right colon cancer  **1** | Admission via emergency department | Intussusception presenting emergently revealing colon cancer | NR |
| **Bawa et al.**  **2023**  **Egypt**  **Nigeria**  **Saudi Arabia** | Retrospective | Colon  Rectum | Risk factors for 30-day postoperative outcomes and in emergency left-sided colorectal operations  **104** | Clinical presentation and Unplanned admission | Malignant colorectal obstruction in 31·7% (33/104) | NR |
| **Uwamariya et al.**  **2022**  **Rwanda** | Retrospective | Colon  Rectum | Epidemiological, pathological and prognostic determinants in patients with CRC  **101** | Clinical presentation | IO rate 18·8% (19/101)  Perforation rate 8·9% (9/101) | NR |
| **Herman et al.**  **2020**  **Tanzania** | Retrospective | Colon  Rectum | Burden, presentation and distribution of CRC  **174** | Unplanned admission | EP-CRC rate 64·9% (111/174)  IO rate 38·5% (67/174) | NR |
| **Abudu et al.**  **2016**  **Nigeria** | Retrospective | Colon  Rectum | Clinical and pathological characteristics of CRC  **45** | Clinical presentation | IO rate 53·3% (24/45) | NR |
| **Sule et al.**  **2011**  **Nigeria** | Prospective | Colon  Rectum | Aetiology, management, morbidity and mortality of adult acute large bowel obstruction  **50** | Clinical presentation | 24% of large bowel obstruction were due to large bowel cancer | NR |
| **Saidi et al. 2008**  **Kenya** | Retrospective | Colon  Rectum | Correlation of clinical data in relation to anatomical location and stage of CRC  **273** | Clinical presentation | IO rate [multiple lesions per patient possible]  Right colon 14·8%,  Left colon 27·1%  Rectum 43·6% | NR |
| **Mahabane et al.**  **2019**  **South Africa** | Case study | Colon | Colon cancer in pregnancy  **1** | Clinical presentation | Obstructing colon tumour in pregnancy | Pregnancy |
| * EP-CRC rate is defined as one or both of the following: -  1. Emergency presentation CRC divided by all CRC diagnosed over the same period  2. Reported by the authors as EP-CRC  † IO rate defined as Intestinal obstruction CRC divided by all CRC diagnosed over the same period  CRC Colorectal cancer  NR Not reported | | | | | | |

Supplemental Table 2: Studies showing EP-CRC Rate *

| **Author** | **Number of cases presenting as emergency** | **Total number of CRC cases** | **EP-CRC rate %** |
| --- | --- | --- | --- |
| Aderibigbe et al. 2024 | 164 | 535 | 30·7 |
| Selemane et al. 2001 | 32 | 62 | 50·8 |
| Dakubo et al. 2010 | 71 | 359 | 19·8 |
| Rahman et al. 2010 | 3 | 36 | 8·3 |
| Uwamariya et al. 2022 | 28 | 101 | 27·7 |
| Herman et al. 2020 | 111 | 174 | 64·9 |
| **TOTAL** | **409** | **1267** |  |
| * EP-CRC Emergency presentation colorectal cancer | | | |

Supplemental Table 3: Studies showing IO rate *

| **Author** | **Number of cases of Intestinal Obstruction** | **Total number of CRC cases** | **IO rate%** |
| --- | --- | --- | --- |
| Abdulkarim et al. 2021 | 65 | 249 | 26·2 |
| Zingeta et al. 2023 | 56 | 209 | 26·8 |
| Moolla et al. 2014 | 203 | 1425 | 14·3 |
| Zeeneldin et al. 2011 | 14 | 159 | 9·0 |
| Iliyasu et al. 1996 | 73 | 526 | 13·9 |
| Ayandipo et al. 2013 | 12 | 61 | 20·0 |
| Ndlovu et al. 2023 | 510 | 2504 | 20·4 |
| Uwamariya et al. 2022 | 19 | 101 | 18·8 |
| Herman et al. 2020 | 67 | 174 | 38·5 |
| Abudu et al. 2016 | 24 | 45 | 53·3 |
| Aderibigbe et al. 2024 | 90 | 535 | 16·7 |
| **TOTAL** | **1133** | **5988** |  |
| * IO Intestinal obstruction | | | |

Supplemental Table 4: Clinical management of emergency presentation colorectal cancer

| Author;  Year of publication | Country | Cancer site | Sample size | Workup | Intervention |
| --- | --- | --- | --- | --- | --- |
| Abdulkarim et al.  2021 | Kenya | Colon  Rectum | 249 | Colonoscopy and biopsy  CT chest and abdomen | NR |
| Ugwu et al.  2001 | Nigeria | Colon  Rectum | 22 | Exploratory laparotomy | Right hemicolectomy (all tumours were right-sided) |
| Herman et al.  2017 | Tanzania | Colon | 1 | Plain abdominal x-ray  Abdominal ultrasound  Full blood count  Erythrocyte sedimentation rate  Renal function, electrolyte levels, hepatic transaminases | Emergency Laparotomy  Sigmoidectomy + double barrel colostomy  Interval colonoscopy + Left hemicolectomy at 6 weeks |
| Saidi et al.  2008 | Kenya | Colon  Rectum | 259 | CT scan  Colonoscopy | NR |
| Zingeta et al.  2023 | Ethiopia | Colon  Rectum | 209 | NR | Emergency surgery 35/104 (33·7%)  Palliative diversion stoma 26 (12·4%) |
| Aderibigbe et al.  2024 | Nigeria | Colon  Rectum | 535 | Clinical assessment  Radiological Imaging  Colonoscopy  Exploratory laparotomy (n=47) 28·7% | Surgery + Chemotherapy 90 (54·9%)  Surgery + Chemotherapy + Radiotherapy 53(32·3%)  Surgery 10 (6·1%)  Chemotherapy 3 (1·8%)  Chemotherapy + Radiotherapy 2 (1·2%)  Surgery + Chemotherapy + Observation 2 (1·2%)  Chemotherapy + Observation 0 (0)  Surgery + Chemotherapy + Radiotherapy +Observation 0 (0)  Surgery + Observation 1 (0·6%)  Unknown 3(1·8%) |
| Selemane et al.  2001 | Mozambique | Colon  Rectum  Anus | 62 | NR | NR |
| Moolla, Z et al.  2014 | South Africa | South Africa | 203 | Clinical assessment, hematologic, and radiologic findings | 166 (82 %) were managed by resection, diversion, or bypass;  37 (18 %) were managed with stent placement |
| Warden et al.  2013 | South Africa | South Africa | 77 | Clinical and radiological evidence of large bowel obstruction  Colonoscopy | Stenting as ‘bridge to surgery’  Stenting as palliation  No surgery |
| Zeeneldin et al.  2011 | Egypt | Colon  Rectum | 159 | NR | NR |
| Iliyasu et al.  1996 | Nigeria | Colon  Rectum | 526 | NR | NR |
| Dakubo et al.  2010 | Ghana | Colon  Rectum | 359 | NR | NR |
| Rahman  2010 | Nigeria | Rectum | 36 | Clinical assessment  Proctoscopy  Sigmoidoscopy  Transrectal ultrasound  Colonoscopy and/or double contrast barium enema | NR |
| Montwedi  2019 | South Africa | Rectum | 1 | Clinical assessment  Routine haematological and biochemical tests  CT scan and MRI for staging | Initially diverting loop colostomy then low anterior resection |
| Soressa et al.  2016 | Ethiopia | Colon  Rectum | 262 | NR | NR |
| Ayandipo et al.  2013 | Nigeria | Rectum  Anus | 61 | NR | Initial faecal diversion then abdominoperineal resection |
| Ndlovu N et al.  2023 | South Africa | Colon  Rectum | 510 | Clinical assessment  Plain radiographs | Resection 370/510 (72·5%)  Diverting colostomy 123/510 (24·1%)  Stent insertion 55/510 (10·8%) |
| Udo IA et al.  2023 | Nigeria | Colon  Rectum | 70 | NR | NR |
| Norman J et al.  2023 | South Africa | Colon  Rectum | 94 | NR | Emergency resection (13 patients)  Non-operative management (1 patient)  Colostomy (4 patients) |
| Ali et al.  2023 | Sudan | Colon | 1 | Clinical assessment  USS abdomen, CT Abdomen, Biochemistry, Carcinoembryonic antigen | Laparotomy + Right hemicolectomy and end-to-end anastomosis |
| Bawa et al.  2023 | Egypt  Nigeria  Saudi Arabia | Colon  Rectum | 104 | NR | Resection depending on location of lesion  “In high-risk patients (septic shock, fecal peritonitis), alternative techniques like terminal ileostomy, Hartmann procedure, bowel bypass, or colostomy were used” |
| Uwamariya et al.  2022 | Rwanda | Colon  Rectum | 101 | NR | NR |
| Herman et al.  2020 | Tanzania | Colon  Rectum | 174 | Colonoscopy 38·6% of study participants.  CT scan 38·5% of study participants | NR |
| Abudu et al.  2016 | Nigeria | Colon  Rectum | 45 | Abdominal USS, sigmoidoscopy, CT scan and occult fecal blood test in 77·6%, 29·9%, 14·9%, and 40·3% of cases respectively.  Colonoscopy was not done for any of the patients. | NR |
| Sule et al.  2011 | Nigeria | Colon  Rectum | 50 | NR | Left hemicolectomy and primary anastomosis (4 patients);  Sigmoid colectomy and primary anastomosis (3 patients);  Low anterior resection and primary anastomosis (3 patients);  Decompressive colostomy (1 patient)  Right hemicolectomy and primary anastomosis (1 patient)  All resections and primary anastomosis involving the right colon were preceded by antegrade on-table colonic lavage  If the left-sided obstructing lesions were thought to be malignant and too advanced to merit any excisional surgery, a biopsy was taken and a decompressive transverse colostomy given, pending the histological confirmation of the diagnosis. |
| Saidi et al.  2008 | Kenya | Colon  Rectum | 26 | NR | NR |
| Mahabane et al.  2019 | South Africa | Colon | 1 | Clinical assessment;  Haemoglobin, HIV test, Syphilis serology  Abdominal USS, Abdominal XRays | Emergency laparotomy, biopsy, and de-functioning colostomy was done  Medical termination of pregnancy  Sigmoid resection and Lymph node biopsy |
| NR Not reported  CT Computed tomography  USS Ultrasound scan | | | | | |

**APPENDIX A**

MEDLINE (via Pubmed) Database search strategy

|  |  |
| --- | --- |
| **Search** | **Query** |
| #1 | **(((colon) OR (rectum)) OR (rectal)) OR (colorectal)** |
| #2 | **(((cancer) OR (carcinoma)) OR (tumour)) OR (malignancy)** |
| #3 | **(((((((emergency) OR (urgent)) OR (acute)) OR (non-elective)) OR (perforation)) OR (peritonitis)) OR (obstruction)) OR (bleeding)** |
| #4 | **(((surgery) OR (operation)) OR (procedure)) OR (resection)** |
| #5 | **Africa** |
| #6 | **#1 AND #2 AND #3 AND #4 AND #5** |

**APPENDIX B**

Reasons for article exclusion at full text stage

| **Authors** | **Year** | **Title** | **Reason(s) for exclusion** |
| --- | --- | --- | --- |
| Ajao OG | 1979 | Colon and anorectal neoplasms in a tropical African population. | Wrong population; full text not accessible |
| Essiet A and Iwatt AR | 1994 | Surgical management of large bowel cancer 1983-1988, University of Calabar Teaching Hospital audit. | Abstract only. Not enough detail to meet study criteria. Full text not accessible. |
| Elmasri SH and Boulos PB | 1976 | Prognosis of colo-rectal carcinoma in Sudan. | Wrong population; full text not accessible |
| Ersumo T and Ali A and Johnson O | 1998 | Cancer of the lower gastrointestinal tract: a five year experience in Ethiopia. | Full text not accessible |
| El-Hennawy MM and Moussa ME and El-Saeidy MK and Shawky AM and Bessa SS and Badour NM | 2003 | Rectal carcinoma in Egyptian patients less than 40 years of age. | Not a research article |
| Madziga AG and Nuhu AI | 2008 | Causes and treatment outcome of mechanical bowel obstruction in north eastern Nigeria. | Wrong population |
| MacKenzie S and Thomson SR and Baker LW | 1992 | Management options in malignant obstruction of the left colon. | Not a primary research article |
| Oueslati A and Ftiriche F and Hajlaoui H and Adala M and Ayachi K and Ben Younes MA | 1991 | Total or subtotal colectomy in the treatment of left, obstructive colon cancers. 4 cases. | Not English |
| Ibrahim OK and Afolayan AE and Adeniji KA and Buhari OM and Badmos KB | 2011 | Colorectal carcinoma in children and young adults in Ilorin, Nigeria. | Wrong population |
| Hassan El Masri S and Khalil T | 1976 | Pattern of intestinal obstruction in Khartoum. | Full text not accessible |
| Ouedraogo S and Tapsoba TW and Bere B and Ouangre E and Zida M | 2019 | Epidemiology, treatment and prognosis of colorectal cancer in young adults in sub-Saharan Africa. | Not English |
| Rouibaa F and Bakkar M and Seddik H and Addioui T and Filali FZ and Akka R and Desla H and Aourarh A | 2013 | Importance of expanded metal prostheses in the management of colonic tumor occlusion: experience of a Moroccan hospital service. | Not English |
| Dhimen, G. and Hami, H. and Boutayeb, S. and Hadrya, F. | 2024 | Colorectal cancer in Morocco: A comprehensive analysis of epidemiological and treatment patterns | Conference abstract only. Not enough detail to meet study criteria. |
| Khougali, H. and Albashir, A. and Daffaalla, H. and Salih, M. | 2019 | Demographic and clinicopathological patterns of colorectal cancer at the National Cancer Institute, Sudan | Wrong population |
| Karrit, S. and Ayari, J. and Fendri, S. and Zribi, A. and Ben Nasr, S. and Balti, M. and Haddaoui, A. | 2019 | Epidemiology, treatment and prognostic factors of colorectal cancers in Tunisia: Compliance with recommendations and therapeutic results | Conference abstract only  No data on EP-CRC |
| Parker, R.K. and Ranketi, S.S. and McNelly, C. and Ongondi, M. and Topazian, H.M. and Dawsey, S.M. and Murphy, G.A. and White, R.E. and Mwachiro, M. | 2019 | Colorectal cancer is increasing in rural Kenya: challenges and perspectives | Wrong population |
| Lahmidani, N. and El Abkari, M. and Ibrahimi, A. and Mellas, N. and El Rhazi, K. and Kharrasse, G. and Afqir, S. and Inrhaoun, H. and Mrabti, H. and Errihani, H. | 2018 | Late diagnosis of colorectal cancer in Morocco : What reasons behind? | Conference abstract only  No data on EP-CRC |
| Belhamidi, M.S. and Kaoukabi, A. and Krimou, H. and Menfaa, M. and Sakit, F. and Choho, K. | 2018 | Colonic lymphoma revealed by ileocecal intussusception in adults: About a case | Not English |
| Heeke, A. and Rashad, N. and Khaled, H.M. and Ali Eldin, N. and Motaal, G.A. and Wang, H. and Weinberg, B. and Salem, M. | 2017 | Variations in clinicopathological features, treatment patterns, and outcomes of young adults with colorectal cancer in the United States and Egypt | Conference abstract only. No data on EP-CRC |
| Abdelkader El Hakim, B. and Yahlali, F. and Yaici, R. and Abada, M. and Aitkaci Ali, S. and Khelladi, A. and Benmouhoub, M. and Smaili, F. | 2015 | Epidemiological and clinical aspects of colorectal cancer: The experience of a single institution in Algeria | Full text not accessible. Not enough detail to meet study criteria. |
| Bekouaci, S. and Smaili, F. and Abada, M. and Benmouhoub, M. and Yaici, R. | 2015 | Clinico-epidemiological profile of colorectal cancer in Algerian patients age 40 and under: Alarming increase in incidence | Full text not accessible. Not enough detail to meet study criteria. |
| Taha, M.O.A. and Abd Abdalla, A.E. and Mohamed, R.S. | 2015 | Pattern & presentation of colorectal cancer in central sudan, a retrospective descriptive study, 2010-2012 | Wrong population |
| Sheikh, A.A. and Joel, A.S. and Johnson, M.A. and Vimalachandran, D. | 2013 | Outcome of colorectal cancer resection in octogenarians | Wrong population; not in Africa |
| Jiménez-Pérez, J; Casellas, J;  García-Cano, J; Vandervoort, J; García-Escribano, O R; Barcenilla, J; Delgado, A A; Goldberg, P; Gonzalez-Huix, F; Vázquez-Astray, E; Meisner, S; | 2011 | Colonic stenting as a bridge to surgery in malignant large-bowel obstruction: A report from two large multinational registries | Wrong population; not in Africa |
| Samia, A. and Karima, O. and Nezar, B. and Hafida, B. and Khawla, D. and Fatima, Z.E. and Samiaziz, B. and Zineb, B. and Khalid, M. and Khalid, A. and Afaf, A. and Sidi, A.I. and Youssra, A. and Omar, E. | 2010 | Primary signet-ring cell carcinoma of the colon and rectum | Wrong population |
| Warden, C. and Stupart, D. and Goldberg, P. | 2010 | Management of left-sided malignant colonic obstruction: An audit of a stent based protocol | Wrong population |
| Mungadi, I.A. | 2001 | Indications and results of right hemicolectomy in Ilorin, Nigeria [5] | Wrong population |
| Beiles, B. | 1981 | Two unusual causes of massive colonic bleeding | Full text not accessible. Not enough detail to meet study criteria. |
| Agyemang-Yeboah F and Yorke J and Obirikorang C and Batu EN and Acheampong E and Frempong EA and Anto EO and Amankwaa B | 2017 | Patterns and presentations of colorectal cancer at Komfo-Anokye teaching hospital Kumasi, Ghana. | Wrong population |
| Hilal, N.A. and Rafei, A. | 2024 | Adult ileocecal intussusception as an unusual presentation of ascending colon adenocarcinoma: a case report from Sudan | Wrong population |
| Aderibigbe, A. and Dare, A. and Knapp, G. and Alatise, O. and Kingham, T.P. | 2021 | Colorectal cancer presentation and survival outcomes in Nigeria: A prospective multi-centre cohort study of 543 patients | Conference abstract only. Subsequent full publication included in review. |
| Gaskill, C. and Gyedu, A. and Agbedinu, K. and Rebollo, D. and Boakye, G. | 2020 | Presentation, treatment, and outcomes of colorectal cancer patients at a referral center in sub-saharan Africa | Wrong population; full text not accessible |
| Belaid, I. and Khechine, W. and Saad, A. and Fki, A. and Makram, H. and El Ghali, A. and Chabchoub, I. and Ezzairi, F. and Ben Fatma, L. and Ben Ahmed, S. | 2019 | Epidemiology, treatment modalities and prognostic factors of colon cancer in the central region of Tunisia | Full text not accessible |
| Driss, A.M. | 2016 | Emergency surgical management of malignat large bowel obstruction | Conference abstract only. Not enough detail to meet study criteria. |
| Alatise, O.I. and Komolafe, A.O. and Famurewa, O.C. and Katung, A.I. and Ariyibi, O.O. and Egberoungbe, A.A. and Olatoke, S.A. and Agodirin, O.S. and Olaofe, O.O. and Kolawole, O.A. and Eaton, A. and Kingham, T.P. | 2016 | Colorectal cancer patient outcomes in Nigeria: Results from the African Colorectal Cancer Group | Conference abstract only. Not enough detail to meet study criteria. |
| Konaté, I; Sridi, A; Ba, P A; Cissé, M; Gaye, M; Ka, I; Touré, F B; Diao, M L; Ka, O; Dieng, M; Dia, A; Touré, C T | 2012 | Descriptive study of colorectal cancer to the surgical clinic of the CHU Aristide le Dantec in Dakar | Not English |
| Kouadio, G.-K. and Turquin, T.-H. | 2003 | Left colonic cancer obstruction in Ivory Coast | Not English |
| EP-CRC Emergency presentation of colorectal cancer | | | |

**APPENDIX C**

Data extraction form

| Publication details | Methodological characteristics | Main findings | Conclusions | Comment(s) |
| --- | --- | --- | --- | --- |
| 1. Paper ID  2. Publication source  (database)  3. Author(s)  4. Year of publication  5. Language | 1. Study design  2. Study objectives  3. Study population  4. Sample size  5. Geographical location/setting | 1. Main outcome(s) evaluated in the study | 1. Conclusions and Recommendations |  |
