# Supplementary material for: Emergency presentation of colorectal cancer in Africa: a scoping review
Source: BMJ Open. 2026 Jan 22;16(1):e110931. doi: 10.1136/bmjopen-2025-110931 (PMC12829401; doi:10.1136/bmjopen-2025-110931)
Supplement: Supplementary file 2 [file bmjopen-2025-110931.reviewer_comments.pdf]

PEER REVIEW HISTORY

BMJ Open publishes all reviews undertaken for accepted manuscripts. Reviewers are asked to complete a checklist review form and are provided with free text boxes to elaborate on their assessment. These free text comments are reproduced below.

ARTICLE DETAILS

Title (Provisional)

Emergency presentation of colorectal cancer in Africa: A scoping review

Authors

Chinyowa, Simbarashe; Murewanhema, Grant; Katsidzira, Leolin; Moodley, Jennifer; Chirenje, Mike; Muguti, Godfrey

---

VERSION 1 - REVIEW

**Reviewer**	**1**
**Name**	**Ros, Javier**
**Affiliation**	**Vall d'Hebron University Hospital, Medical Oncology**
**Date**	**10-Oct-2025**
**COI**	**None**

Dear Authors,

I have reviewed your article regarding emerging-presentation colorectal cancer (EP-CRC) with great interest. You have conducted a thorough review to investigate EP-CRC in Africa, and your effort is commendable. Unfortunately, due to the lack of available publications and detailed data, it is difficult to draw a clear conclusion, and the final message remains somewhat limited.

Moreover, the definition of EP-CRC itself is not standardized — different clinical presentations and varying definitions are used, which complicates appropriate data collection and interpretation. Consequently, the reported rates of EP-CRC vary widely across countries.

From my perspective, despite the obvious limitations due to the scarcity of information in the literature, your research represents a significant effort. While it may not be expected to produce robust statistical results, it successfully raises awareness about the urgent need to implement effective screening programs in Africa, which could potentially improve clinical outcomes on the continent.

Minor comments.

1.- Some tables (like table 1) are actually large. I would suggest moving this table as supp material while keeping table 2 and table 3 that summarize the most relevant results.

2.- Table 1: some abbreviations must be defined such as "NR" or "EP"

3.- Table 4: i would suggest adding each study sample size.

**Reviewer**	**2**
**Name**	**Li, Jian**
**Affiliation**	**the Third People's Hospital of Mianyang, Department of general surgery**
**Date**	**14-Nov-2025**
**COI**	**None**

This review is the first of its kind to systematically map the evidence on EP-CRC in Africa. The topic is highly relevant given the increasing burden of colorectal cancer in low- and middle-income countries and the lack of organized screening programs in Africa. The authors have successfully highlighted a critical gap in the literature and provided a foundation for future research and policy-making. The focus on emergency presentations—a marker of health system performance—is both innovative and clinically meaningful. Below are my comments on the manuscript:

1.The discussion rightly identifies the variability in how "emergency presentation" is conceptualized across the included studies (e.g., based on timing of admission, route of admission, or clinical picture). This is a critical methodological point that significantly impacts the interpretation and comparability of the reported EP-CRC rates. To make this crucial information immediately accessible to the reader and to provide a more robust foundation for the discussion, I strongly recommend the addition of a new column to Table 1 with the header: "Definition of Emergency Presentation". For each study, briefly summarize the definition used. For example: "Unplanned admission" ,"Admission via emergency room", "Based on clinical presentation (e.g., obstruction)", "Not explicitly defined", and so on.

2.The inclusion of full article titles makes the table unnecessarily wide and visually cluttered. Removing them will create a more compact and reader-friendly layout, allowing the more critical data (e.g., study design, cancer site, EP-CRC rate) to be more easily compared across studies.

3.The data presented in Table 2 (EP rate) and Table 3 (IO rate) are already contained within the "Main findings" column of Table 1. Presenting them again in separate tables is redundant and unnecessarily lengthens the manuscript.

4.I recommend moving all explanatory notes regarding superscripts/subscripts (e.g., *) and abbreviations (e.g., NR=Not reported) from the table titles and the table body into footnotes for each respective table.

5.I recommend a thorough revision of the manuscript to standardize the use of abbreviations throughout the text and tables. Including: multiple definitions for the same abbreviation; abbreviations defined but not used consistently;

6.Using a descriptive format like "Author, Year, Country" within the narrative text is not the conventional way to cite scientific literature. For example, "In a study in Nigeria, Aderibigbe, 2024 described risk factors for EP-CRC." proposed revision as "In a study in Nigeria, Aderibigbe et al. identified several risk factors for EP-CRC."

VERSION 1 - AUTHOR RESPONSE

Response to Reviewer 1

Thank you very much for taking the time to review our manuscript. Your reflection on our work is greatly appreciated. We have addressed your specific comments in the table below.

**Comment**	**Response**
1.- Some tables (like table 1) are actually large. I would suggest moving this table as supp material while keeping table 2 and table 3 that summarize the most relevant results.	Data in Table 1 has been summarized to make it more compact e.g. we have removed the column with publication Title in line with a suggestion by Reviewer 2. To avoid redundant data, we have moved Table 2 and 3 to supplemental material.
2.- Table 1: some abbreviations must be defined such as "NR" or "EP"	We have defined all abbreviations as footnotes at the bottom of the table.
3.- Table 4: i would suggest adding each study sample size.	We have added each study sample size to Table 4. Table 4 has been renamed Table 2 because we have moved the original Table 2 and Table 3 to the supplementary data section.

Response to Reviewer 2

We greatly appreciate your time and expertise in going through our manuscript. Your detailed feedback has helped to highlight areas needing clarification and improvement. We have addressed each of your comments in the table below.

**Comment**	**Response**

1.The discussion rightly identifies the variability in how "emergency presentation" is conceptualized across the included studies (e.g., based on timing of admission, route of admission, or clinical picture). This is a critical methodological point that significantly impacts the interpretation and comparability of the reported EP-CRC rates. To make this crucial information immediately accessible to the reader and to provide a more robust foundation for the discussion, I strongly recommend the addition of a new column to Table 1 with the header: "Definition of Emergency Presentation". For each study, briefly summarize the definition used. For example: "Unplanned admission" ,"Admission via emergency room", "Based on clinical presentation (e.g., obstruction)", "Not explicitly defined", and so on.	We have created a column in Table 1 “Definition of Emergency Presentation” under which we have summarized the definition used in each study .
2.The inclusion of full article titles makes the table unnecessarily wide and visually cluttered. Removing them will create a more compact and reader-friendly layout, allowing the more critical data (e.g., study design, cancer site, EP-CRC rate) to be more easily compared across studies.	We have removed full article titles from table 1 and summarized the data as far as possible to make it more compact and allow easier readability.
3.The data presented in Table 2 (EP rate) and Table 3 (IO rate) are already contained within the "Main findings" column of Table 1. Presenting them again in separate tables is redundant and unnecessarily lengthens the manuscript.	We had extracted data in table 2 (EP rate) and Table 3 (IO rate) in an attempt to make clear how we calculated an average EP rate and IO rate. However, we acknowledge and appreciate your concern about data redundancy and manuscript length. We have therefore, moved these tables to the supplementary data section (Supplemental Table 1 and Table 2, respectively).
4.I recommend moving all explanatory notes regarding superscripts/subscripts (e.g., *) and abbreviations (e.g., NR=Not reported) from the table titles and the table body into	We have moved all explanatory notes to the footnote section of each table.

footnotes for each respective table.	
5.I recommend a thorough revision of the manuscript to standardize the use of abbreviations throughout the text and tables. Including: multiple definitions for the same abbreviation; abbreviations defined but not used consistently;	We have revised the entire manuscript to ensure standardized use of abbreviations throughout the text and tables.
6.Using a descriptive format like "Author, Year, Country" within the narrative text is not the conventional way to cite scientific literature. For example, "In a study in Nigeria, Aderibigbe, 2024 described risk factors for EP-CRC." proposed revision as "In a study in Nigeria, Aderibigbe et al. identified several risk factors for EP-CRC."	We have revised all citations within the manuscript to conform to the recommended format.
